# Improving the Mechanical Properties and Microstructure of 12 mol% Ceria-Stabilized Tetragonal Zirconia Polycrystal Ceramics with Low-Content Nd_2_O_3_

**DOI:** 10.3390/ma17225426

**Published:** 2024-11-06

**Authors:** Zengqing Sun, Xiaoyu Li, Jinxin Xing, Min Gan, Zhiyun Ji, Yong Lyu

**Affiliations:** 1 School of Minerals Processing & Bioengineering, Central South University, Changsha 410083, China; 2 School of Metallurgical and Ecological Engineering, University of Science and Technology Beijing, Beijing 100083, China

**Keywords:** 12Ce-TZP-xNd_2_O_3_, mechanical properties, microstructure

## Abstract

In this study, 12 mol% ceria-stabilized tetragonal zirconia polycrystal ceramics with xNd_2_O_3_ (where x equals 0, 0.1, 0.2, 0.3, 0.4, 0.5, and 0.7) were synthesized via the solid-state method, and the effects of Nd_2_O_3_ doping amounts on the mechanical properties and microstructure were studied. The results show that with an increase in the Nd_2_O_3_ doping amount, the grain size of the ceramics was reduced from 2.93 μm to 0.69 μm. The hardness and strength of the ceramics increased significantly, while the fracture toughness decreased. The reduction in fracture toughness was attributed to the reduction in tetragonal grain size, which suppressed the tetragonal–monoclinic phase transformation caused by stress. Additionally, as the content of Nd_2_O_3_ increased, the formation of cubic zirconia accelerated, but no second phase was observed. Most importantly, when the doping amount of Nd_2_O_3_ reached 0.3 mol%, the comprehensive mechanical characteristics of the ceramics were optimal. This provides a research basis for the preparation of nanoscale 12 mol% ceria-stabilized tetragonal zirconia polycrystal ceramics.

## 1. Introduction

Over the recent decades, zirconia (ZrO_2_) has become a prevalent material in structural ceramics due to its superior mechanical attributes [1,2,3,4,5], including its robust strength and exceptional fracture toughness. ZrO_2_ ceramics have three different crystal structures and can exist in three distinct crystallographic forms: the monoclinic phase (m-ZrO_2_), the tetragonal phase (t-ZrO_2_), and the cubic phase (c-ZrO_2_). The three crystal structures can be transformed into each other at different temperature ranges. High temperatures yield the c-ZrO_2_ phase, middle temperatures yield the t-ZrO_2_ phase, and low temperatures yield the m-ZrO_2_ phase. t-ZrO_2_ remains stable at temperatures exceeding 1200 °C, and this temperature is lower than the threshold required for transformation from a tetragonal to a monoclinic structure [6,7,8].

Zirconia exists in a monoclinic phase (m) at room temperature. When monoclinic-phase zirconia is heated to 1170 °C, it transforms into tetragonal-phase zirconia, and this transformation is accompanied by a 7–9% volume shrinkage. The transformation rate is fast. In the cooling process, tetragonal zirconia transforms into monoclinic zirconia at 950 °C, and this is called martensitic transformation. In this phase transformation process, there is a volume expansion of 3–5%, which makes the ceramic materials easy to crack and they lose their application value, so zirconia ceramics cannot be used in practice. To maintain the tetragonal phase of ZrO_2_ at room temperature, a widely adopted approach is the incorporation of stabilizing agents such as yttrium oxide (Y_2_O_3_) and cerium oxide (CeO_2_) [9]. These additives help to preserve the desired crystal structure under normal environmental conditions. The 12 mol% ceria-stabilized tetragonal zirconia polycrystal (12Ce-TZP) ceramics have long been popular because of their excellent fracture toughness and aging resistance [10,11,12,13,14,15,16,17,18,19,20] compared with Y-TZP. Nevertheless, the mechanical strength and hardness of Ce-TZP are somewhat insufficient. This is primarily attributed to the influence of CeO_2_, which encourages the acceleration of grain growth and an increase in grain size during the sintering process. These properties are not conducive to the material’s intended applications. The ceramic properties of Ce-TZP can be enhanced by incorporating additional phases like Al_2_O_3_ and MgAl_2_O_4_. This incorporation leverages the principles of dispersion strengthening and fine grain strengthening to bolster the material’s mechanical properties. The synthesis and sintering behaviors of CeO_2_-ZrO_2_ were studied by Duh [21]. The results showed that 12 mol% CeO_2_ is sufficient to stabilize ZrO_2_ in the tetragonal phase when synthesizing CeO_2_-ZrO_2_ by the coprecipitation method. Almost completely dense 12Ce-TZP ceramics can be obtained after calcination for 6.5 h at 1400 °C. The addition of 1 mol% Y_2_O_3_ and 2 mol% MgO into the CeO_2_-ZrO_2_ system can reduce the grain size of sintered samples and form a single tetragonal structure in 10CeO_2_-ZrO_2_ lattices. However, the mechanical strength and hardness of 12Ce-TZP are subdued in comparison to the 3Y-TZP counterpart, which constrains its industrial application. Acknowledging the limitations of 12Ce-TZP ceramics, a series of 12Ce-TZP-xNd_2_O_3_ ceramics were synthesized via a solid-state reaction approach. This study meticulously examined the impact of Nd_2_O_3_ incorporation on both the mechanical attributes and the microstructural development of the 12Ce-TZP ceramic system [22,23,24,25].

## 2. Experimental Procedure

In this experiment, ceramics were synthesized via the solid-state synthesis method. Raw materials of ZrO_2_, CeO_2_, and Nd_2_O_3_ (Aladdin, Shanghai, China, purity ≥ 99.99%, 60 ± 10 nm) were weighed and calcined in a muffle furnace for 3 h at 800 °C to eliminate the water that had been absorbed by the raw materials. The raw materials were then mixed in a ball mill jar in the following chemical proportions: based on the weighed ZrO_2_, 12 mol% of CeO_2_ was added. After that, different amounts of Nd_2_O_3_ were introduced as a dopant, with specific doping levels of 0, 0.1, 0.2, 0.3, 0.4, 0.5, and 0.7 mol%. Following this, a suitable volume of anhydrous ethanol along with zirconia balls was introduced, and the mixture was ground for a duration ranging from 10 to 15 h, during which, the ball mill speed was increased. The required ratio of raw materials was milled on a ball mill at 650 r/min for 24 h. Afterwards, the milled slurry was poured into a high-energy ball mill for 3 h at a milling speed of 2200 r/min. Then, the milled slurry was dried in an electric constant temperature drying oven. The dried powder was ground in an agate bowl and sieved through an 80-mesh sieve to obtain the final powder. The final sample compact was obtained via pressing and pre-forming with 8 MPa and cold isostatic pressing with 200 MPa. The formed sample was placed in a high-temperature resistance furnace and calcined for 3 h at 1450 °C to obtain the required ceramic sample. Finally, each sample was characterized and its mechanical properties were tested.

To examine the cross-sectional morphology of the samples, a field emission scanning electron microscope (FE-SEM, GeminiSEM 500, ZEISS, Oberkochen, Germany) was utilized. Additionally, we employed Energy-Dispersive Spectroscopy (EDS, EDS QUANTAX, Bruker, Germany) to quantitatively map individual elements within specific regions. The selection of these regions was guided by Backscattered Electron (BSE) imaging, which provided a clear contrast based on the chemical composition of the samples. This contrast enhancement facilitated the identification of areas with distinct elemental compositions. For the EDS analysis, an acceleration voltage of 20 kV was used to ensure accurate and reliable elemental mapping.

The phase structure of the 12Ce-TZP ceramics was identified using X-ray diffraction (XRD, X’Pert PRO MPD, PANalytical, Almelo, The Netherlands). For the XRD analysis, a copper target was employed and operated at a voltage of 40 kV and a current of 20 mA. The scan was conducted over a range of 20° to 80° at a rate of 0.02 s per degree. The density of the ceramic samples was measured using the specific gravity bottle method, following the principle of Archimedes. Additionally, Raman spectroscopy (LabRam HR Evolution, HORIBA Scientific, Paleso, France, excited by a He-Ne laser at 532 nm) was used to measure phase variables near the indentation for various doping levels.

Measurements of Vickers hardness of the ceramic samples were performed by the XHV-1000 Automatic Turret Digital Display Micro-Vickers Hardness Meter (Cossim, Beijing, China). We used a load of 50 kg, holding time of 20 s, and 10 pressing points for each ceramic sample and then averaged the measured results. The fracture toughness of the ceramic specimens was evaluated through an indentation method, while the three-point bending strength was determined using a PT-307 device (Precise Test, Dongguan, China) for measuring flexural strength. For these tests, each sample set comprised 10 individual samples. The testing parameters included a loading speed of 0.2 mm/min, with the samples having a thickness of 3 mm, width of 4 mm, and support span of 60 mm. The calculations for the fracture toughness were based on the following formula:

(1) $$\sigma =\frac{3FL}{2bh^{2}}$$

where (*σ*) denotes the flexural strength measured in megapascals (MPa), (*F*) signifies the applied load, (*L*) represents the supported span between the two points, (*b*) indicates the breadth of the specimen, and (*h*) corresponds to the thickness of the specimen.

## 3. Results and Discussion

**Figure 1:**
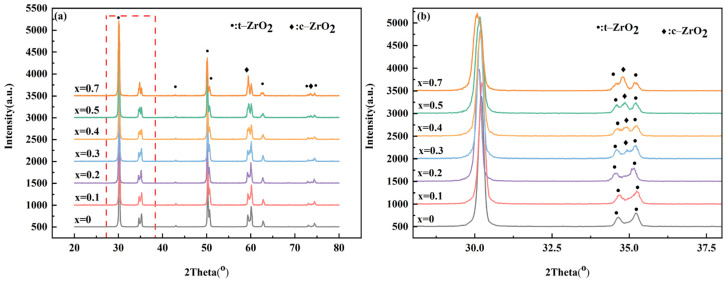
XRD patterns of the 12Ce-TZP-xNd_2_O_3_ samples: (**a**) original image, (**b**) enlarged view.

As depicted in Figure 1a, the X-ray diffraction patterns of the 12Ce-TZP-xNd_2_O_3_ (where x equals 0, 0.1, 0.2, 0.3, 0.4, 0.5, and 0.7) ceramics did not exhibit the characteristic peaks of the monoclinic m(−111) and m(111) phases at 28.5° and 31°. These peaks are typically associated with the monoclinic phase and were absent compared to the standard diffraction peaks of tetragonal zirconia (PDF card no. 17-0923). Figure 1b shows a local enlargement of 2θ = (28–38°). Evidently, when the molar percentage of Nd_2_O_3_ doping reached 0.3 mol%, the characteristic peaks indicative of the cubic phase emerged at 2Theta = 35 degrees. These were in alignment with the standard diffraction peaks at 2Theta = (71–76 degrees) for the tetragonal phase, specifically, t(004), t(400), t(200), and t(002), and also included some distinctive peaks for the cubic phase, namely, c(400) and c(200). These findings indicated that an increase in the cubic phase content influenced the mechanical attributes of the ceramic materials [26].

Additionally, the XRD results in Figure 1 show that all patterns were characteristic of the ZrO_2_ phase structure, with no second phase appearing due to Nd_2_O_3_ doping. Studies have indicated that as the content of Nd_2_O_3_ is increased, the content of cubic zirconia gradually increases, proving that Nd_2_O_3_ completely dissolves into the zirconia lattice. In our previous research [27], it was shown that with multi-element doping of ZrO_2_ at low doping levels, the second-phase doping elements can completely dissolve into the zirconia lattice.

**Figure 2:**
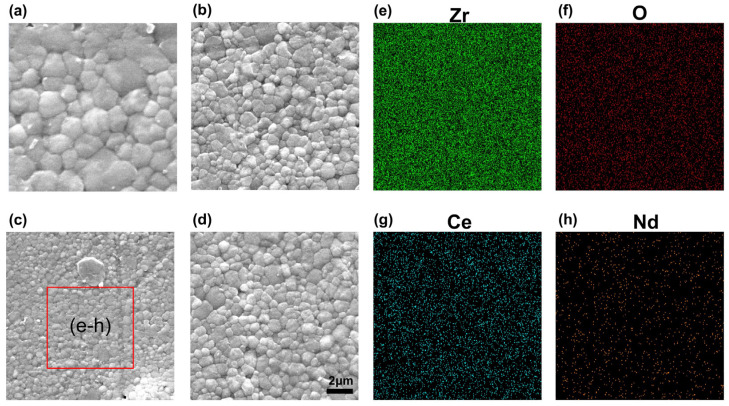
FE-SEM of the surface morphology of 12Ce-TZP-xNd_2_O_3_ ceramics (**a**) x = 0, (**b**) x = 0.1, (**c**) x = 0.3, and (**d**) x = 0.7. Local EDS element distribution of (**e**–**h**) corresponding to (**c**) x = 0.3.

Figure 2 shows the surface microstructure of different Nd_2_O_3_ doping amounts, with a grain size of 2.93 μm for undoped Nd_2_O_3_ ceramics. As the doping amount of Nd_2_O_3_ increased, the grain size of the ceramics in this system gradually decreased and became more uniform. This observation was consistent with previous studies that demonstrated the grain-growth-inhibiting effect of Nd_2_O_3_ doping in ceramics, such as the work by Xu et al., who reported that Nd_2_O_3_ doping could effectively inhibit grain growth in tetragonal zirconia polycrystalline ceramics [28].

Figure 2e,f show the local EDS element distribution with x = 0.3 mol%. The EDS results showed that the content of Nd_2_O_3_ was very low, further indicating that even minor amounts of Nd_2_O_3_ doping could significantly influence grain growth. The results in Table 1 show the density and grain size of all the ceramics. When the doping amount of Nd_2_O_3_ was 0.3 mol%, the minimum grain size of the ceramics reached 690 nm.

**Figure 3:**
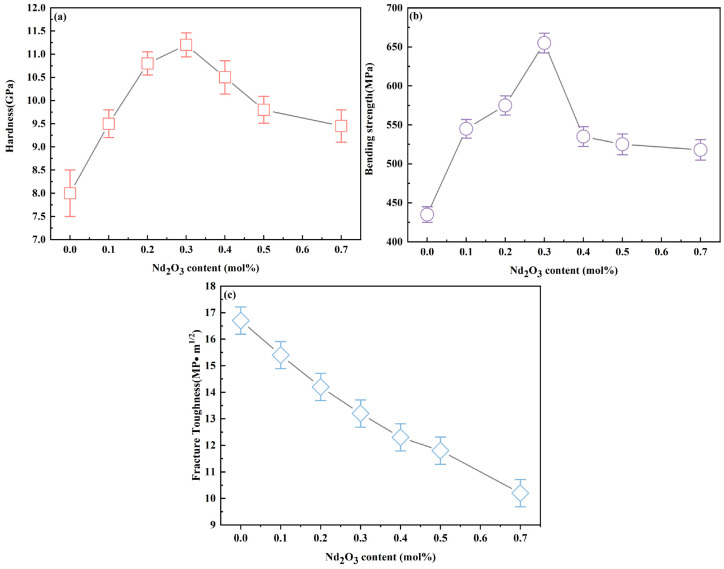
Mechanical properties of 12Ce-TZP-xNd_2_O_3_ ceramics: (**a**) hardness, (**b**) strength, (**c**) fracture toughness.

Figure 3a shows the hardness of the 12Ce-TZP-xNd_2_O_3_ ceramics. The hardness of the ceramics increased from 8 GPa when the doping amount of Nd_2_O_3_ was x = 0 mol% to 11.2 GPa when the doping amount of Nd_2_O_3_ was x = 0.3 mol% and then decreased with the increase in the doping amount of Nd_2_O_3_. Figure 3b shows the strength and hardness curves of the ceramic specimens. When the doping content of Nd_2_O_3_ reached 0.3 mol%, the maximum strength reached 655 MPa. Figure 3c shows that the fracture toughness of the ceramics decreased from 16.7 MPa·m^1/2^ to 13.2 MPa·m^1/2^ with the increase in Nd_2_O_3_ content. This enhancement of hardness and strength with Nd_2_O_3_ doping contrasted with the properties of the undoped 12Ce-TZP ceramics prepared by Maleki et al., which had a hardness of 7.83 GPa and strength of 210 MPa, indicating the beneficial effect of Nd_2_O_3_ addition on the mechanical properties of the ceramics [29].

Generally speaking, the grain size of 12Ce-TZP was relatively coarse. Currently, it is widely believed that doping with Al_2_O_3_ or MgAl_2_O_4_ can form a second phase and precipitate at the grain boundary, inhibiting the grain growth of 12Ce-TZP and improving its mechanical properties [14,15,16,17,18]. However, the doping amount of Al_2_O_3_ or MgAl_2_O_4_ needs to be large, and achieving a uniformly distributed second phase is not easy. Zhang studied the effect of La_2_O_3_ doping on 3Y-TZP [19,20]. When positive ions such as Nd^3+^, which had a larger radius than the Zr^4+^ ions, were doped, strong segregation occurred at the grain boundary of ZrO_2_, ultimately improving the thermal stability and mechanical properties of the material. Providing that the doping level of Nd_2_O_3_ was maintained within an appropriate limit, it could positively influence the microstructure and mechanical properties of TZP ceramics. As illustrated in Figure 3, the impact of Nd_2_O_3_ on the hardness and strength of the samples was evident. Studies have demonstrated that the hardness and strength of transformation-toughened zirconia ceramics follow a similar pattern, which is in agreement with the observed experimental outcomes here [25,30]. It was evident that the hardness and strength of the samples exhibited a regular change with varying concentrations of Nd_2_O_3_ doping. Up to a doping level of 0.3 mol%, there was a noticeable increase in both hardness and strength. This enhancement was believed to be due to the role of Nd^3+^ as a tetragonal zirconia stabilizer, which reduced the phase transformation capability of zirconia as its concentration increased. As previously discussed, the ionic radius of Nd^3+^ exceeds that of Zr^4+^. In addition, Nd_2_O_3_ could not only dissolve completely but could also form an additional cubic phase when the doping content was high. Therefore, the strength and hardness of the samples doped with 0.3 mol% Nd_2_O_3_ were higher than those of the samples without doping, which was related to the mechanism of solution strengthening. Once the concentration of Nd_2_O_3_ doping surpassed 0.3 mol%, a marginal reduction in hardness was detected, while there was a significant drop in strength. This decrease was attributed to the emergence of both cubic and monoclinic phases within the material’s structure. Therefore, for the samples doped with more than 0.3 mol% Nd_2_O_3_, the significant decrease in strength was closely related to the increase in the cubic phase compared with the mechanical properties. According to the above analysis, the specimens doped with 0.3 mol% Nd_2_O_3_ had good mechanical properties.

In order to further explain the reduction of fracture toughness, the following formula was introduced [11,12]:

(2) $${∆G}_{t-m}={∆G}_{c}+{∆G}_{se}+{∆G}_{s}$$

where *G_c_* represents the change in chemical free energy, *G_se_* is the change in strain free energy, and *G_s_* is the change in surface free energy. It was well known that the addition of yttrium oxide and cerium oxide could improve the *Gc*, and the increase in *G_se_* could improve the *G_s_* by increasing the elastic modulus of the matrix and reducing the grain size. The doping of cerium oxide increased the chemical free energy, while the doping of Nd_2_O_3_ inhibited the growth of grains, thus enhancing the surface free energy, inhibiting tetragonal–monoclinic phase transformation and reducing the fracture toughness, which provided a basis for the phase transformation toughening mechanism.

For the ceramic materials, the reduction in grain size could effectively improve the strength of the ceramics, which was attributed to the fine grain strengthening mechanism [31]. Research by Sarkar et al. indicated a linear relationship between the strength and grain size of the material [32]. The reduction in grain size led to more complex and tortuous ceramic grain boundaries, absorbing more fracture energy and improving the strength. Furthermore, as evidenced by the XRD data presented in Figure 1, there was a decline in the hardness and strength of the ceramic system once the doping level surpassed 0.3 mol%. This reduction was linked to the introduction of the cubic phase within the material’s structure [33]. For partially stable ZrO_2_ ceramics, the existence of a stress-induced phase transition mechanism made the fracture toughness of the ceramics more complex. According to Formula (2), we predicted that the decrease in grain size suppressed tetragonal–monoclinic phase transformation, hindered the stress-induced phase transition mechanism, and reduced the fracture toughness of the ceramic system. Therefore, we verified this by indentation experiments and measured the phase variables near the indentation with different doping amounts using Raman spectroscopy; the formula was as follows [34]:

(3) $$V_{m}=\frac{I_{m}^{178}+I_{m}^{188}}{0.32\left(I_{t}^{148}+I_{t}^{260}\right)+I_{m}^{178}+I_{m}^{188}}$$

where *I_t_* and *I_m_* are the integral intensities of the Raman band and *V_m_* is the volume fraction of the monoclinic phase.

**Figure 4:**
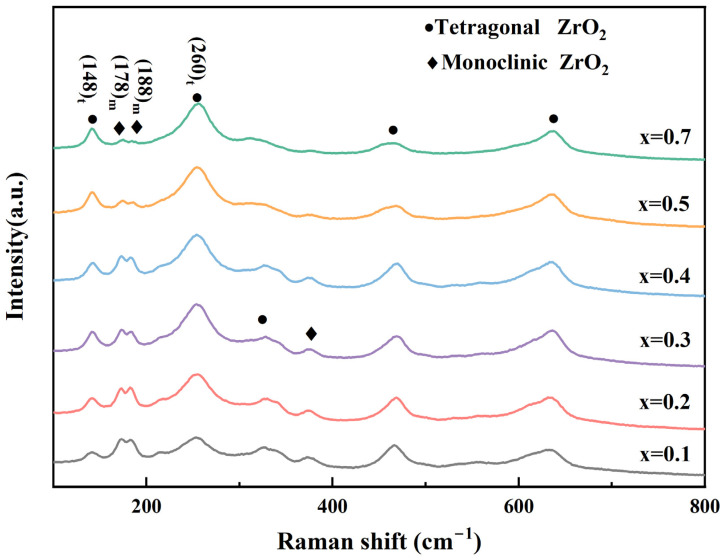
Raman spectrum of 12Ce-TZP-xNd_2_O_3_ ceramics.

The Raman spectra corresponding to the tetragonal and monoclinic phases in the 12Ce-TZP-xNd_2_O_3_ ceramics are shown in Figure 4. According to Formula (3), the phase content of tetragonal to monoclinic phases at different locations could be calculated.

**Figure 5:**
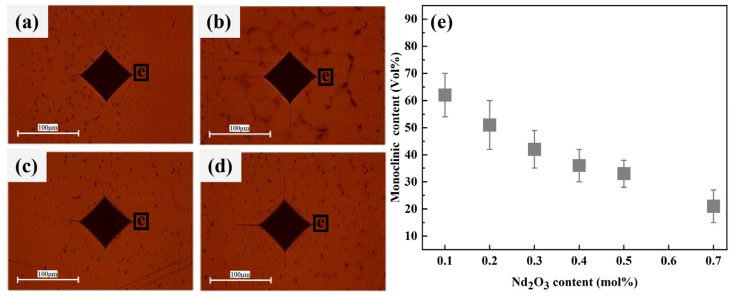
Indentations of 12Ce-TZP-xNd_2_O_3_ ceramics: (**a**) x = 0, (**b**) x = 0.1, (**c**) x = 0.3, (**d**) x = 0.7. (**e**) Average value of the monoclinic phase content corresponding to the selected region.

**Figure 6:**
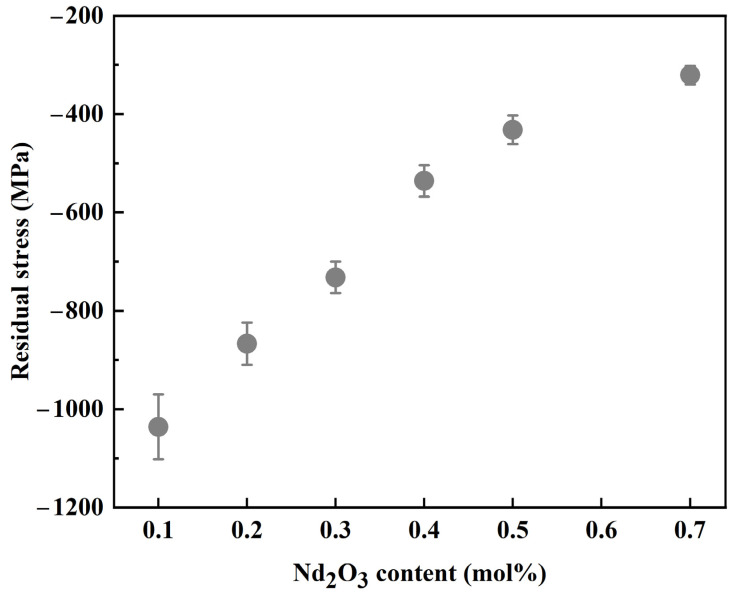
Average residual stress in the selected area around the 12Ce-TZP-xNd_2_O_3_ ceramic indentation.

In addition, based on previous research, the stress distribution near indentations with different doping levels was also analyzed through Raman spectroscopy; the formula was as follows [35]:

(4) $$\sigma =-3.826\;ln\left(\frac{\left|9.32-∆V\right|}{9.56}\right)$$

where ∆V is the 468 cm^−1^ shift and σ is the residual stress. Figure 5 shows the indentations of 12Ce-TZP-xNd_2_O_3_ ceramics, along with the average values of phase variables obtained from the corresponding selected areas. It was observed that as the grain size decreased, the phase variables at the selected area of the indentation in Figure 5e decreased, and the decrease in fracture toughness was also attributed to the decrease in tetragonal–monoclinic phase transformation. In addition, it was seen that the cracks around the indentation of the undoped 12Ce-TZP ceramics were significantly shorter, which was linearly positively correlated with the phase variables. Previous literature has reported that the grain size of the tetragonal phase is larger, with greater fracture toughness [27]. We knew that the fracture toughness of 3Y-TZP ceramics was much lower than that of 12Ce-TZP ceramics, which could be proven. Figure 6 shows the average residual stress in the corresponding phase transformation selection area. The stress caused the tetragonal–monoclinic phase transformation to generate significant compressive stress around the indentation, which suppressed the propagation of indentation cracks, thereby achieving the goal of improving fracture toughness. The distribution of compressive stress was linearly related to the phase variable, which further proved that the larger the tetragonal phase grain, the greater the fracture toughness.

## 4. Conclusions

Due to the low hardness and low strength of 12Ce-TZP ceramics, 12Ce-TZP-xNd_2_O_3_ ceramics were prepared by the solid-phase method. The mechanical properties and microstructures of the 12Ce-TZP-xNd_2_O_3_ ceramics were as follows:

1. The doping of Nd_2_O_3_ reduced and homogenized the grain size of the ceramics, which was due to the segregation of large trivalent cations on the grain boundary, thus inhibiting the growth of grains. Additionally, as the content of Nd_2_O_3_ increased, the formation of cubic zirconia accelerated, but no second phase was observed.
2. Because of the appearance of the cubic phase, with the increase in Nd_2_O_3_ doping content, the hardness and strength of the ceramics first increased and then decreased, which reduced the mechanical properties of the ceramics. The decrease in fracture toughness was attributed to the reduction in tetragonal grain size, which suppressed the tetragonal–monoclinic phase transformation caused by stress, thereby reducing the toughening mechanism of phase transformation.
3. When the doping amount of Nd_2_O_3_ reached 0.3 mol%, the comprehensive mechanical properties of the 12Ce-TZP-xNd_2_O_3_ ceramics reached the optimum levels (hardness: 11.2 GPa, strength: 655 Mpa, fracture toughness: 16.7 MP m^1/2^).

## Figures and Tables

**Table 1:** Grain size and bulk density of 12Ce-TZP-xNd_2_O_3_ ceramics.

Samples	Average Grains (μm)	Density (g·cm^3^)
12Ce-TZP	2.93 ± 0.15	6.026
12Ce-TZP-0.1Nd_2_O_3_	1.62 ± 0.11	6.031
12Ce-TZP-0.2Nd_2_O_3_	1.12 ± 0.08	6.022
12Ce-TZP-0.3Nd_2_O_3_	0.69 ± 0.04	6.034
12Ce-TZP-0.4Nd_2_O_3_	0.95 ± 0.03	6.031
12Ce-TZP-0.5Nd_2_O_3_	0.98 ± 0.032	6.024
12Ce-TZP-0.7Nd_2_O_3_	1.03 ± 0.032	6.036

## Data Availability

The data presented in this study are available on request from the corresponding author due to privacy that prevent public dissemination.
